# Supplementary material for: HIVprotI: an integrated web based platform for prediction and design of HIV proteins inhibitors
Source: J Cheminform. 2018 Mar 9;10:12. doi: 10.1186/s13321-018-0266-y (PMC5845081; doi:10.1186/s13321-018-0266-y)
Supplement: Supplementary file 2 — Additional file 2. Source code of HIVProtI web server. [file 13321_2018_266_MOESM2_ESM.zip › HIVprotI_Source-code/hivprotI/weka-3-6-6/doc/weka/gui/ResultHistoryPanel.RMouseAdapter.html]

ResultHistoryPanel.RMouseAdapter


---


|  |  |  |
| --- | --- | --- |
| **Overview** \| **Package** \| **Class** \| **Tree** \| **Deprecated** \| **Index** \| **Help** \| **Weka's home** | |  |
| **PREV CLASS**   **NEXT CLASS** | **FRAMES**    **NO FRAMES**     **All Classes** |  |
| SUMMARY: NESTED \| FIELD \| CONSTR \| METHOD | DETAIL: FIELD \| CONSTR \| METHOD |  |


---


## weka.gui Class ResultHistoryPanel.RMouseAdapter

```
java.lang.Object
  java.awt.event.MouseAdapter
      weka.gui.ResultHistoryPanel.RMouseAdapter
```

**All Implemented Interfaces:**: java.awt.event.MouseListener, java.awt.event.MouseMotionListener, java.awt.event.MouseWheelListener, java.io.Serializable, java.util.EventListener

**Enclosing class:**: ResultHistoryPanel

---

``` public static class ResultHistoryPanel.RMouseAdapter extends java.awt.event.MouseAdapter implements java.io.Serializable ```

Extension of MouseAdapter that implements Serializable.

**See Also:**: Serialized Form

---

**Constructor Summary**

`ResultHistoryPanel.RMouseAdapter()`


**Method Summary**

**Methods inherited from class java.awt.event.MouseAdapter**

`mouseClicked, mouseDragged, mouseEntered, mouseExited, mouseMoved, mousePressed, mouseReleased, mouseWheelMoved`

**Methods inherited from class java.lang.Object**

`equals, getClass, hashCode, notify, notifyAll, toString, wait, wait, wait`

**Constructor Detail**

### ResultHistoryPanel.RMouseAdapter

```
public ResultHistoryPanel.RMouseAdapter()
```


---


|  |  |  |
| --- | --- | --- |
| **Overview** \| **Package** \| **Class** \| **Tree** \| **Deprecated** \| **Index** \| **Help** \| **Weka's home** | |  |
| **PREV CLASS**   **NEXT CLASS** | **FRAMES**    **NO FRAMES**     **All Classes** |  |
| SUMMARY: NESTED \| FIELD \| CONSTR \| METHOD | DETAIL: FIELD \| CONSTR \| METHOD |  |


---
